# Novel insights into amylin aggregation

**DOI:** 10.1080/13102818.2014.901680

**Published:** 2014-05-28

**Authors:** Karen Pillay, Patrick Govender

**Affiliations:** ^a^ Department of Biochemistry, School of Life Sciences, University of KwaZulu-Natal, Durban, South Africa

**Keywords:** amylin, nanoparticle tracking analysis, surface plasmon resonance, type II diabetes

## Abstract

Amylin is a peptide that aggregates into species that are toxic to pancreatic beta cells, leading to type II diabetes. This study has for the first time quantified amylin association and dissociation kinetics (association constant (*k_a_*) = 28.7 ± 5.1 L mol^−1^ s^−1^ and dissociation constant (*k_d_*) = 2.8 ± 0.6 ×10^−4^ s^−1^) using surface plasmon resonance (SPR). Thus far, techniques used for the sizing of amylin aggregates do not cater for the real-time monitoring of unconstrained amylin in solution. In this regard we evaluated recently innovated nanoparticle tracking analysis (NTA). In addition, both SPR and NTA were used to study the effect of previously synthesized amylin derivatives on amylin aggregation and to evaluate their potential as a cell-free system for screening potential inhibitors of amylin-mediated cytotoxicity. Results obtained from NTA highlighted a predominance of 100–300 nm amylin aggregates and correlation to previously published cytotoxicity results suggests the toxic species of amylin to be 200–300 nm in size. The results seem to indicate that NTA has potential as a new technique to monitor the aggregation potential of amyloid peptides in solution and also to screen potential inhibitors of amylin-mediated cytotoxicity.

## Abbreviations

*A*β: amyloid *beta*
*AFM*: atomic force microscopy
*DLS*: dynamic light scattering
*DMF*: dimethylformamide
*DMSO*: dimethylsulfoxide
*ESI-QTOF*: electrospray ionization time-of-flight spectroscope
*FTIR*: Fourier transform infrared
*HEPES*: 2-(2-hydroxyethyl)-1-piperazineethanesulfonic acid
*HFIP*: hexafluoroisopropanol
*HPLC*: high performance liquid chromatography
*NTA*: nanoparticle tracking analysis
*NMR*: nuclear magnetic resonance
*PrP*: prion protein
*SPR*: surface plasmon resonance
*STEM*: scanning transmission electron microscopy
*TFA*: trifluoroacetic acid
*ThT*: thioflavin T

## Introduction

Full-length human amylin (amylin) is a 37 amino acid long peptide which is released together with insulin from pancreatic *beta* cells.[1,2] Accumulation of amylin can result in its soluble monomeric form aggregating into toxic oligomers and eventually fibrils,[3–6] allowing it to be classified as an amyloidogenic peptide and implicating it in the development of type II diabetes.[2,7–10] Inhibition or prevention of amylin aggregation and subsequent cytotoxicity has always relied on compounds that could bind to amylin,[11–16] and numerous studies have investigated the multi-faceted dynamics of amylin aggregation. Techniques that have been used to elucidate the conformational change from a random coil or helical structure to a β-sheet structure and to provide a model for the secondary structure of amylin include circular dichroism (CD) spectroscopy,[17,18] Fourier transform infrared (FTIR) spectroscopy,[19] two-dimensional (2D) spectroscopy,[20,21] and solid-state nuclear magnetic resonance (NMR).[22] Filtration assays [4,18] were used to monitor the time-dependent change in aggregate size but does not allow for real-time monitoring of the aggregation process. For observation of the aggregation process in real time, atomic force microscopy (AFM) [23,24] can be employed, which generates quantitative data on the diameter as well as the growth rate of amylin aggregates. The latter mentioned amylin aggregation dynamics were also elucidated using scanning transmission electron microscopy (STEM).[17,25,26] Two other techniques that have been used to monitor amylin aggregation dynamics include electrochemical analysis,[27] which is based on the oxidation of tyrosine; and tryptophan triplet quenching,[28] which as its name implies monitors the quenching of the triplet state of tryptophan by cysteine or disulphides. Although no quantitative data were presented, these techniques were used to study the rate of interaction between the chain termini of amylin and the kinetics of amylin aggregation respectively. A more recent study made use of the thioflavin T (ThT) dye and total internal reflection fluorescence microscopy to visualize amylin aggregation.[29] Although AFM and STEM data can be combined to generate association kinetics, none of these techniques were independently capable of generating quantitative data on the association and dissociation kinetics of amylin. In addition, no study to date has monitored the change in size of aggregates that formed from unconstrained amylin in solution over real time.

From as early as 1994, surface plasmon resonance (SPR) technology has been used to determine the aggregation kinetics of amyloidogenic proteins.[30–32] SPR can monitor protein–protein interaction and is based on the principle that the refractive index at a surface changes proportionally to the amount of molecules present on it, which can be measured using an optical system.[33] Some of the advantages of SPR are that it allows for fibril growth to be monitored over minutes or even seconds, very low sample concentrations are required, and no peptide-labelling strategy is necessary, thus permitting direct analysis of unmodified peptide sequences.[34] Moreover, quantitative data can be generated to express the rate of association as well as the dissociation kinetics.

Of all the amyloidogenic proteins, amyloid *beta* (Aβ) interactions have been most extensively studied using SPR [31,34–51] followed by prion protein (PrP), which have been implicated in Alzheimer's disease and transmissible spongiform encephalopathy (Prion diseases), respectively.[52–55] Initially, Myszka et al. [56] reported SPR as a suitable technique to assess the association and dissociation kinetics of Aβ aggregation. Thereafter, SPR was employed to extensively characterize the aggregation kinetics of Aβ,[34,39] and an SPR-based assay was subsequently developed to allow identification of small molecules that bind to Aβ and which could act as potential therapeutic agents against Alzheimer's disease.[35] It was also reported that SPR could be used as a potential assay for screening anti-prion molecules.[53] For more details with respect to SPR investigations into Aβ aggregation, an extensive review by Aguilar and Small [57] is recommended. However, up until now, SPR-based studies into amylin aggregation are limited to the attachment of biotinylated-amylin derivatives to strepavidin-coated sensor chips.[58,59]

Jaikaran et al. [58] evaluated the interaction of rat amylin and compounds present in the secretory granule of pancreatic *beta* cells such as insulin, somtostatin and proinsulin, with the sensor chip-bound biotinylated-amylin. A similar SPR-based approach was employed by Wei et al. [59] and in both of these studies, it was suggested that insulin inhibits the formation of β-sheet structures by binding to biotinylated-amylin.[58,59] The most recent study immobilized nanoparticles on a sensor chip and used SPR to evaluate the binding affinity of amylin for these particles.[60] However, the generated data were not indicative of the kinetics of amylin association and dissociation. In addition, an SPR-based strategy is yet to be evaluated as a potential cell-free selection system for inhibitors of amylin-mediated cytotoxicity.

Elucidation of the aggregation dynamics of amylin could also involve monitoring the change in size of the amylin aggregates. As mentioned earlier, other studies that have monitored the size of amylin aggregates made use of STEM which involves adsorption of aggregates onto copper grids or AFM which involves growing aggregates on mica surfaces.[4,17,18,25,26] Although these studies provided valuable insight into amylin aggregate structures, they did not allow for unconstrained real-time monitoring of amylin aggregation. In addition, it has been observed that fibrils formed from unconstrained amylin in solution exhibit distinctly different morphologies from those propagated on a mica surface.[23,24] It was suggested that the mica surface used in AFM could possibly impede coiling of fibrils around each other and thereby prevent formation of higher order fibrils.[23,24]

Another commonly used particle sizing technique which does not involve attachment of the molecule to a solid support is dynamic light scattering (DLS). This technique is easy to perform and has been proven to produce accurate results in a short time.[61] DLS relies on the phenomenon that Brownian motion of particles causes fluctuations in their scattered light intensity which is proportional to the particle size. An alternative technique that makes use of the Brownian motion of particles for size determination is nanoparticle tracking analysis (NTA) which was recently developed by Malloy and Carr.[62] The NTA technology makes use of a laser light scattering microscope and charge-coupled device (CCD) camera that visualizes and records nanoparticles. Thereafter, NTA-based software is able to track nanoparticles that move under Brownian motion and relate the movement to its size using a formula (Equation (1)) as derived by Filipe et al. [63] from the Stokes–Einstein equation [64] as follows:(1) 
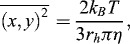
where *k_B_* is the Boltzmann constant, and 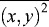 is the mean-squared speed of a particle at temperature *T*, in a medium of viscosity η with a hydrodynamic radius of *r_h_*. Thus, NTA can estimate the size of particles in a poly-disperse sample and was shown to be a suitable technique for monitoring protein aggregation.[63]

To date, DLS has been used to size the monomeric form of amylin and the aggregated form of amylin derivatives.[65,66] However, it should be mentioned that DLS and NTA technologies are yet to be employed to evaluate the oligomeric and fibrillar forms of amylin.

It is also noteworthy that cell-free systems such as CD,[13,15,16,67,68] FTIR spectroscopy,[13] transmission electron microscopy,[12,13,15,67–70] the ThT assay [12,15,16,69–72] and sedimentation assays [12,16,68] cannot differentiate between the toxic oligomeric and non-toxic fibrillar forms of amylin and thus are used together with cell-based assays for screening of potential inhibitors of amylin-induced cytotoxicity. However, cell-based systems can be time-consuming and extremely expensive as it is dependent on the growth rate of a particular cell line and is also labour-intensive, thus highlighting the need for a cell-free system for efficient routine screening of potential inhibitors of amylin-mediated cytotoxicity.

In the present study, we employ DLS, NTA and SPR strategies to evaluate the aggregation dynamics of amylin. Moreover, the potential of these techniques as a new screening technology for inhibitors of amylin-mediated cytotoxicity was assessed by monitoring the effect of chemically synthesized human amylin derivatives on amylin–amylin interaction and correlating results to previously published cytotoxicity data.[73] NTA was also used to identify the size of the amylin aggregates that are probably responsible for amylin-mediated cytotoxicity.

## Materials and methods

### Reagents

All 9-fluorenylmethoxycarbonyl (Fmoc) protected amino acids and coupling reagents were purchased from GLS Biochem Systems, Inc. (China). The following protecting groups were used for the side chains of the amino acids: trityl (Trt) for asn, cys, gln and his; t-butyl ether (tBu) for ser and thr; 2,2,4,6,7-pentamethyl-dihydrobenzofuran-5-sulfonyl (Pbf) for arg; and t-butyloxycarbonyl (Boc) for lys. The PAL-ChemMatrix resin was purchased from Matrix Innovation (Canada) and all solvents for synthesis and purification were of high performance liquid chromatography (HPLC) grade and were purchased from Sigma–Aldrich (USA). The CM5 sensor chip and the thiol coupling kit were purchased from BIAcore AB (Sweden).

### Peptide synthesis

**Figure 1.**
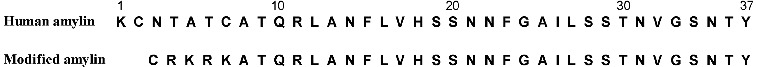
Peptide sequences of chemically synthesized full length human amylin and modified amylin (MA).

For the SPR experiment, a modified version of human amylin (MA) was synthesized, wherein the first seven residues at the *N*-terminal of human amylin were replaced by the linker sequence CRKRK (Figure 1). This modification contains a cysteine residue at the *N*-terminus to enable thiol coupling to the BIAcore sensor chip, thus allowing MA to be used as the ligand for the SPR-based experiment. Chemically synthesized human amylin (amylin) [74] was used as the analyte for the SPR experiments, as well as for the DLS and NTA experiments.

Modified amylin was synthesized on a 0.1 mmol scale, using a CEM microwave peptide synthesizer as described previously.[74] Briefly, deprotection was performed using 20% piperidine in dimethylformamide (DMF). The activator used in the synthesis was 0.5 mol L^−1^ 2-(1H-benzotriazole-1-yl)-1,1,3,3-tetramethyluronium hexafluorophosphate (HBTU) in DMF, with 1 mol L^−1^ *N*,*N*-diisopropylethylamine (DIPEA) in DMF serving as the activator base. The peptide was cleaved from the resin using 5% tri-isopropylsilane in trifluoroacetic acid (TFA) (v/v) for 2 h.

Amylin derivatives (Table 1) were synthesized as potential inhibitors of amylin-mediated cytotoxicity as previously reported [73] and their impact on amylin aggregation dynamics was investigated.

**Table 1.** Sequences of chemically synthesized non-methylated and *N*-methylated amylin derivatives.

Amylin segment	Non-methylated sequence	*N*-methylated sequence
Amylin_3–6_	NTAT (n1^#^)	NTAT* (m1^#^)
Amylin_9–13_	TQRLA (n2^#^)	TQRLA* (m2^#^)
Amylin_15–20_	FLVHSS (n3^#^)	FLVHSS* (m3^#^)
Amylin_22–27_	NFGAIL (n4^#^)	NFGAIL* (m4^#^)
Amylin_29–34_	STNVGS (n5^#^)	STNVGS* (m5^#^)

^#^n1–n5 and m1–m5 are shorthand notations that are used to denote amylin derivatives.

*N-methylated amino acids are underlined.

### Peptide purification

The modified amylin was purified on an ACE C18 preparative column (250 mm × 22 mm, Scotland) as previously described.[74] A dual-buffer system was employed, with TFA serving as the ion-pairing agent. The first buffer consisted of 0.1% TFA/H_2_O (v/v) whilst the second buffer was composed of 0.1% TFA/acetonitrile (v/v). The peptides were eluted using a gradient of 0%–90% of 0.1% TFA/acetonitrile (v/v) over 90 min with a flow rate of 20 mL min^−1^. The solvent from pooled peptide-containing fractions was evaporated to 20 mL and the samples were snap-frozen in liquid nitrogen and lyophilized.

### Peptide analysis

The purified peptide was analysed with an Agilent 1100 HPLC system fitted with a Waters XBridge C18 column, 250 mm × 3.6 mm as previously described.[74] Chromatography was performed over 90 min, using a gradient of 0%–90% buffer B at a flow rate of 0.3 mL min^−1^ and the eluent was monitored at a UV wavelength of 215 nm. A Bruker electrospray ionization time-of-flight spectroscope (ESI-QTOF) in positive mode was used to obtain mass spectra (MS) and matrix assisted laser desorption ionization time-of-flight mass spectroscopy (MALDI-TOF MS) was performed with an Autoflex III instrument (Bruker) operated in positive mode with cyano-4-hydroxycinnamic acid being used as the matrix.

### Disaggregation method

Disaggregation of amylin and its derivative MA were performed as previously described.[73] Pre-weighed amylin samples were solubilized in 200 μL hexafluoroisopropanol (HFIP):TFA solution (50:50, v/v), sonicated for 10 min and left overnight. The solvents were then removed under vacuum, using a centrifugal evaporator for approximately 1–2 h. Approximately 100 μL HFIP was added to the amylin, followed by vortexing and the solvent was removed by rotary evaporation for 1–2 h. To remove all traces of TFA, the latter process was repeated twice using HFIP (100 μL).

### Amylin immobilization for surface plasmon resonance (SPR)

All sensorgrams for SPR were performed on a BIAcoreX biosensor and analysed using BIAevaluation version 4.1.1 software (BIAcore AB, Sweden). The running buffer for all SPR-based experiments was adapted from Jaikaran et al. [58] and contained 10 mmol L^−1^ 4-(2-hydroxyethyl)-1-piperazineethanesulfonic acid (HEPES), 4 mmol L^−1^ EDTA, 150 mmol L^−1^ NaCl, 0.005% Tween 20, and 5% dimethylsulfoxide (DMSO), pH 7.4. The following solutions were adapted from Liu et al. [75] and Takahashi et al. [50] for the regeneration/washing steps. Solution 1 contained 6 mol L^−1^ guanidine hydrochloride (Gdn-HCl) in 10 mmol L^−1^ Tris-HCl (pH 8.0), solution 2 was made up of 5% DMSO in water (v/v), and solution 3 was composed of 100 mmol L^−1^ NaOH. Attachment of MA to the CM5 sensor chip followed standard ligand thiol coupling conditions as suggested by the manufacturer. A flow rate of 10 μL min^−1^ was maintained for all immobilization steps, unless otherwise stated. The carboxymethyl dextran matrix on the CM5 sensor chip was activated by injecting 40 μL of a 1:1 mixture of 200 mmol L^−1^ *N*-ethyl-*N’*-[(dimethylamino)propyl]-carbodiimide (EDC) and 50 mmol L^−1^ *N*-hydroxysuccinimide (NHS), followed by the addition of 40 μL of 80 mmol L^−1^ 2-(2-Pyridinyldithio)-ethaneamine hydrochloride (PDEA) in 50 mmol L^−1^ sodium borate buffer (pH 8.5). A 20 μL aliquot of 1 μmol L^−1^ MA contained in 10 mmol L^−1^ sodium acetate buffer (pH 5.0) was then injected into the activated flow cell. An aliquot (40 μL) of 50 mmol L^−1^ L-cysteine in 0.1 mol L^−1^ sodium acetate and 1.0 mol L^−1^ NaCl (pH 4.0) was then injected to eliminate free, unreacted maleimide groups so as to prevent non-specific binding. To remove non-covalently associated MA, the surface of the chip was washed sequentially with 15 μL of each of the three wash solutions described above whilst maintaining a flow rate of 20 μL min^−1^. The control flow cell was set up using the protocol described above with elimination of the MA attachment step and the three subsequent wash steps. Curve fitting of each data set at varying concentrations was performed using BIAevaluation version 4.1.1 software.

### Surface plasmon resonance analysis of amylin and its derivatives

A flow rate of 5 μL min^−1^ was maintained for all subsequent SPR experiments, unless otherwise stated. To determine amylin aggregation kinetics, a concentration series (40 μmol L^−1^ to 120 μmol L^−1^) of disaggregated amylin samples were solubilized in filter sterilized (0.22 μmol L^−1^, nylon) running buffer by sonication for 5 min and injected onto the sensor chip-immobilized amylin for 3 min. Dissociation kinetics was monitored for 360 s by allowing running buffer only to pass over the sensor chip surface. Whilst maintaining a flow rate of 20 μL min^−1^, regeneration was achieved using 15 μL of solution 1 and solution 3, respectively. Triplicate injections of 40, 50, 60, 80 and 120 μmol L^−1^ disaggregated amylin were analysed in a random order with two regeneration steps being performed between each concentration.

SPR was also performed to evaluate the effect of each amylin derivative on amylin–amylin interaction and hence to determine if this technique could be used as an efficient cell-free assay to screen for potential inhibitors of amylin-mediated cytotoxicity. Stock solutions (1 mmol L^−1^) of each derivative were prepared in running buffer and each amylin derivative was then added to lyophilized samples of disaggregated amylin to yield a final ratio of 50 μmol L^−1^:250 μmol L^−1^ (amylin:amylin derivative). Mixtures of amylin and each of the amylin derivatives were sonicated for 5 min before 15 μL of each sample was injected onto sensor chip-immobilized amylin. Dissociation was once again monitored for 360 s by allowing running buffer to flow over the sensor chip surface. Between each sample, regeneration was performed as described above. All sensorgrams were double referenced, i.e. responses were corrected with both blank buffer injections and the response from the reference flow cell. Double referencing was performed to remove system artefacts as recommended by Myszka.[56] For samples containing a mixture of amylin and each of its derivatives, responses were also corrected with sensorgrams obtained from 15 μL injections of samples containing each amylin derivative only (250 μmol L^−1^).

### Dynamic light scattering (DLS)

DLS experiments were performed in a Zetasizer NanoZS (Malvern Instruments Ltd., United Kingdom). To monitor the change in size of amylin aggregates during aggregation, a sample of disaggregated amylin was solubilized by sonication for 5 min in filter sterilized 10 mmol L^−1^ sodium phosphate buffer, pH 7.4 containing 50 mmol L^−1^ NaCl (Buffer A) to a final concentration of 50 μmol L^−1^ and analysed at various time points. To determine if lower concentrations of the sample affected the poly-dispersity index, serial dilutions of the 50 μmol L^−1^ amylin sample were prepared in Buffer A and analysed. Zetasizer version 6.30 software (Malvern Instruments Ltd., United Kingdom) was used to analyse and convert data sets obtained to apparent hydrodynamic diameters.

### Nanoparticle tracking analysis (NTA)

NTA measurements were performed with a NanoSight LM20 instrument (NanoSight, United Kingdom) equipped with a 640 nm laser and a temperature controlled sample chamber. To monitor the change in size of amylin aggregates during aggregation, disaggregated amylin was solubilized by sonication for five minutes in Buffer A to a final concentration of 50 μmol L^−1^. The amylin sample was then injected into the sample chamber and allowed to equilibrate to 37 °C (approximately 1 min) before 60 s video recordings were captured at 10 min intervals for the first hour and then at 24 h. The sample temperature was maintained at 37 °C for the entire duration of the experiment. These experimental conditions were similar to that used for a previously reported cytotoxicity experiment,[73] thus allowing correlation of results obtained from the two techniques. To assess the effect of the amylin derivatives on amylin aggregation, stock solutions (1 mmol L^−1^) of each amylin derivative were prepared in Buffer A. Each amylin derivative was then added to lyophilized samples of disaggregated amylin to give a final ratio of 50 μmol L^−1^:250 μmol L^−1^ (amylin:amylin derivative). Samples containing amylin and each of its derivatives were sonicated for 5 min before being injected into the sample chamber. Once again, samples were maintained at 37 °C and 60 s video footage were recorded every 10 min for the first hour and then at 24 h. All videos were captured with the single shutter and gain modes. Analysis was performed using NanoSight NTA version 2.2 software with a viscosity setting of 0.70. All determinations were performed in duplicate with standard deviations displayed as error bars on the NTA graphs.

### Statistical analysis

In this study, the means and standard deviations were employed in the unpaired *t-*test to statistically compare the concentration of 100–150, 150–200 and 200–300 nm aggregates in samples containing amylin only to that formed in samples containing a mixture of amylin and each of its derivatives. GraphPad InStat version 3 for Windows XP (GraphPad Software, USA) was used for the statistical analysis and results were considered significantly different if *p*-values were less than 0.05.

## Results and discussion

**Figure 2.**
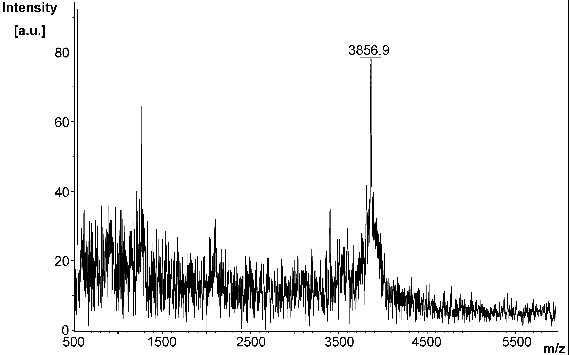
MALDI-TOF spectrum of modified amylin (MA).

As observed by MALDI-TOF analysis (Figure 2), MA was synthesized with high purity and a yield of 9% (35 mg). The linker sequence CRKRK in MA was selected and incorporated since the basic R-groups of the arginyl and lysinyl residues will repel each other under physiological conditions. Upon binding of MA to the sensor chip for SPR-based experiments, this linker region ensures that the 8–37 region of amylin will be a sufficient distance away from the surface of the chip and from each other, thereby allowing it to freely interact with the analyte. The linker sequence in MA replaced the 1–7 region of amylin since the 8–37 region alone was previously shown to form fibrils with the same morphology as fibrils formed from full length amylin that exhibited a typical amyloidogenic β-sheet structure.[17,76] In addition, solid-state NMR models have revealed that the 1–7 region of amylin is not involved in the formation of β-sheet structures.[21,22,76] Moreover, a peptide analogue of the 8–37 region of amylin exhibited fibrillogenesis kinetics that was comparable to full length amylin.[77] Thus, it was strategized that MA would have a similar binding affinity as full length amylin thereby being suitable to study amylin–amylin interactions.

Whilst the kinetics of Aβ interactions are known, only limited data regarding human amylin-based aggregation are available. Although previous microscopy-based studies (AFM and STEM) have reported on the size of amylin oligomers, there currently exists no information with respect to the magnitude of free fibrillar structures formed by amylin in solution. The data presented herein shed some light on the above-mentioned amylin parameters thereby promoting a greater understanding of amylin aggregation, a potential causative agent of type II diabetes.

**Figure 3.**
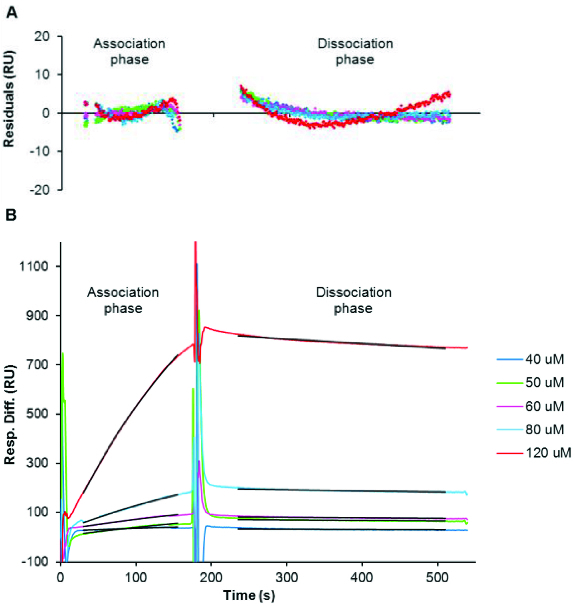
Kinetic analysis of amylin aggregation as generated from SPR-based experiments. (A) Depicts the residuals of curve fitting, i.e. the difference between the observed and calculated values for association and dissociation of amylin. Sensorgram plots (B) of various concentrations (40–120 μmol L^−1^) of disaggregated amylin that were injected for 3 min to observe association and with dissociation being monitored for 6 min whilst maintaining a flow rate of 5 μL min^−1^. The black lines represent the global fit for association (30–155 s) and dissociation (235–510 s). The χ^2^ = 3 for association; χ^2^ = 3.7 for dissociation.

When disaggregated amylin was immobilized, a change of 640 response units (RUs) was recorded, indicating that amylin attachment to the sensor chip was efficient since a similar immobilization density was observed during previous SPR-based kinetic studies of Aβ.[39] Once this was established, further experiments were performed to determine the kinetic rate constants of amylin aggregation. Samples of disaggregated amylin at various concentrations (40 μmol L^−1^ to 120 μmol L^−1^) were analysed and global fitting of the data using different types of interactions was performed using BIAevaluation version 3.1 software to determine the kinetic rate constants which include the association constant (*k_a_*) and dissociation constant (*k_d_*). The three sets of data were fitted independently to verify reproducibility and it should be noted that all three replicates followed a similar trend (representative plot in Figure 3).

The 1:1 Langmuir interaction (Equation (2)) was found to fit the generated data very well, since the curves generated from this type of interaction fit the observed plots very well (representative residual plots and fitted curves in Figure 3). This type of interaction could thus give the best representation of the kinetic data generated for amylin. In this interaction, one ligand (L) molecule interacts with one analyte (A) molecule and the resultant complex (LA) follows pseudo first-order kinetics. The rate (see Equation (3)) indicates that the amount of complex formed over time is proportional to *k*_a_ and *k*_d_ in the presence of excess analyte.[78,79] As suggested by the BIAevaluation handbook and also observed from the residual plots in Figure 3(A), the χ^2^ values are less than 10, which is an acceptable value for good fitting of data. This confirms an alignment of the observed and expected data since they are within the noise range of the sensorgrams[78]:(2) 
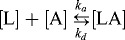

(3) 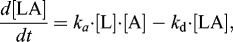
where L is immobilized ligand, A is analyte, LA is ligand–analyte complex and *t* is time.

The observed amylin association constant (*k*_a_) of 28.7 L mol^−1^ s^−1^ ± 5.1 L mol^−1^ s^−1^ can be interpreted as 0.035 complexes of amylin being formed per second in a one molar solution of amylin. An observed amylin dissociation constant (*k*_d_) of 2.8 × 10^−4^ s^−1^ ± 0.6 × 10^−4^ s^−1^ suggests that under experimental conditions, it takes approximately 60 min for the complete dissociation of free amylin from sensor chip-immobilized amylin.

Previous quantitative studies using STEM postulated that the mass-per-length (MPL) of an amylin protofibril is 10 kDa nm^−1^ [26] and AFM studies have shown that amylin grows at a rate of 1.1 nm min^−1^.[23] From these studies by Goldsbury et al.,[23] it can be estimated that the approximate rate of amylin aggregation is 11 kDa min^−1^. As indicated by SPR-generated data of this study, 0.035 complexes of amylin form per second which translates to 2.1 complexes being formed per minute. This implies an aggregation rate of 8.2 kDa min^−1^ (wherein a single amylin molecule of 3.9 kDa is added per complex). The SPR-derived amylin association kinetics generated in this study seemingly correlate to that previously reported by Goldsbury et al.,[23,26] who employed a similar strategy of pre-binding amylin to a solid support and monitoring association kinetics of amylin that is free in solution.

Since amylin and Aβ share similar structural properties of amyloidosis,[4] it is opportune in this study to compare their association and dissociation kinetics. Aβ kinetics generated from previous SPR-based experiments revealed an association rate of 0.01 complexes per second.[39] Global fitting of the SPR data also found that Aβ dissociation follows the first-order kinetic model with a *k_d_* of 2.23 × 10^−3^ s^−1^.[39] This was further supported by a recent SPR-based Aβ study that determined a *k_a_* of 0.01 complexes per second for Aβ association following first-order kinetics, whilst the *k_d_* was 9.2 × 10^−4^ s^−1^ ± 1.3 × 10^−4^ s^−1^.[80] It is noteworthy that the former SPR-based evaluation of Aβ was performed under similar conditions to those used in the present study.[39] Both SPR-based studies on Aβ kinetics have established that fewer complexes of Aβ are formed over time.[39,80] In addition, Aβ was revealed to have a faster dissociation time than amylin (7.5 and 18 min versus 60 min).[39,80]

The data presented herein complements previously published association kinetics of amyloid aggregation whilst differences were observed in the dissociation kinetics. This could indicate that even though amylin and Aβ share similarities in their conformational properties of aggregation, having non-identical sequences could result in different interactions being responsible for stabilizing the aggregated structure, thereby accounting for differences in their dissociation kinetics. According to a model developed by Petkova et al.,[81] the 12-21 and 30-40 regions of the Aβ peptide form the β-sheet structure whilst the 22-29 region is responsible for forming the β-strand turn. These regions contain predominantly hydrophobic amino acids and it has been suggested that the only hydrophilic interaction could be present between the oppositely charged side chains of asp and lys, thereby implying that only hydrophobic interactions are responsible for stabilizing the structure of aggregated Aβ.[22,81] In contrast, NMR studies on amylin propose that there are hydrophobic interactions between the 15–17 and 23–27 regions of amylin whilst inter-chain hydrophilic (electrostatic) interactions could be present between the 28–31 regions of amylin.[22] It is thus probable that both hydrophobic and hydrophilic interactions contribute to stabilization of the β-sheet structure of amylin and since hydrophilic interactions are stronger than hydrophobic interactions, this could most likely account for the longer dissociation time of amylin as observed in the present study. In addition, the SPR-derived aggregation kinetics of Aβ that have been described above were generated using the 1–40 form of Aβ which was previously shown to aggregate much more slowly than the 1–42 form of Aβ.[82]

In a previous study, we reported on methylated and non-methylated derivatives of amylin as potential inhibitors of *in vitro* amylin-mediated cytotoxicity.[73] However, SPR-generated data on the effect of these amylin derivatives on amylin–amylin interactions could not be correlated to the previously reported inhibitory activities of these amylin derivatives.[73] In support of this, a similar observation was reported by Lee et al. [83] who found that increased binding of a molecule to Aβ does not necessarily indicate that the molecule has a capacity to reduce cytotoxicity. In addition, even though insulin has been reported to inhibit fibril formation, SPR-based experiments recorded a change of only 15 RUs during binding of insulin to sensor chip-bound biotinylated amylin.[58] It is thus suggested that SPR under the experimental conditions employed in this study cannot be used as a technique to evaluate potential inhibitors of amylin-mediated cytotoxicity.

Hence, both DLS and NTA strategies, which allow for real-time monitoring of aggregation dynamics in solution, were subsequently employed to evaluate the effect of these amylin derivatives on the size of amylin aggregates formed over a 24-h period. DLS analysis of various concentrations of disaggregated amylin alone yielded no results since reports generated by the Zetasizer version 6.30 software recorded a poly-dispersity index of greater than 0.7, indicating that the sample is very poly-disperse. Thus, the limitation of DLS to accurately size amylin aggregates appears to stem from the inherent principle of the technique which is based on the concept that the intensity of light scattered from particles is proportional to its size. Since larger particles will scatter more light than smaller particles, small particles will be obscured by large ones and thus DLS will not be able to accurately size particles in a poly-dispersed sample.[63] Our observation is also supported by light scattering data on the amyloidogenic proteins Aβ [84] and prion protein,[85] both of which have also shown that these samples were poly-disperse.

As an alternative technique, NTA was employed to evaluate the size of amylin aggregates that form in real time when disaggregated amylin samples are unconstrained in solution. Initial NTA of a 50 μmol L^−1^ amylin sample established that a total concentration of 3.12 × 10^8^ aggregates per millilitre was present, which is within the ideal concentration range (1 × 10^8^ to 25 × 10^8^ aggregates per millilitre) for accurate NTA.[86] This concentration was thus selected for all further NTA experiments.

**Figure 4.**
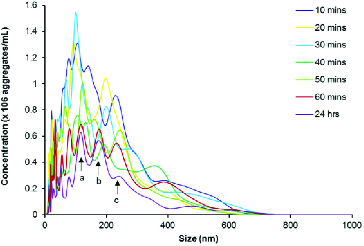
NTA size distribution profile of disaggregated amylin (50 μmol L^−1^) in 10 mmol L^−1^ sodium phosphate buffer, pH 7.4 containing 50 mmol L^−1^ NaCl. The sample was maintained at 37 °C for the duration of the experiment. Video recordings (duration of 60 s) for NTA were taken at each time point, using the single shutter and gain mode. Arrows labelled a, b and c indicates the predominant size range over 24 h.

**Figure 5.**
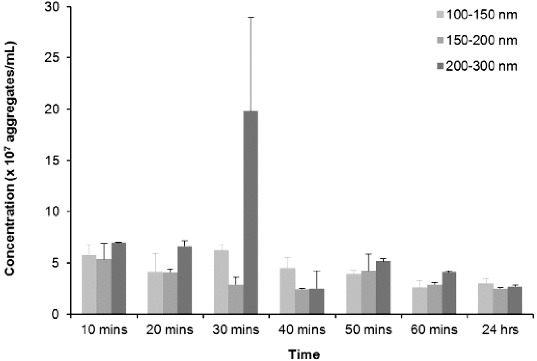
NTA distributions of 100–150 nm, 150–200 nm and 200–300 nm aggregates that formed over time from disaggregated amylin (50 μmol L^−1^) in 10 mmol L^−1^ sodium phosphate buffer (pH 7.4) containing 50 mmol L^−1^mM NaCl. Samples were maintained at 37 °C for the duration of the experiments. Video recordings (duration of 60 s) for NTA were taken at each time point, using the single shutter and gain mode.

Taking resuspension, injection and equilibration times into consideration, the time lapse from addition of buffer to analysis was approximately 10 min, making analysis of a zero time-point impractical. Figures 4 and 5 illustrate that aggregates are present at the first 10-min time-point, which seems to be consistent with previous reports using other technologies that have demonstrated almost instantaneous aggregation of amylin.[18,87,88] The three major peaks indicate that the predominant aggregate sizes are within the following size ranges: 100–150 nm, 150–200 nm and 200–300 nm (Figure 4). During the first 50 min, peaks in the range 100–200 nm are not well defined, indicating that there is a range of aggregates from 100 to 200 nm. As time progresses, the amylin aggregates clearly resolve into two distinct peaks that are indicated by arrows a and b (Figure 4). Further analyses on these size ranges were performed and are depicted in Figure 5. During the first hour, the number of 100–150, 150–200 and 200–300 nm aggregates decreased by more than 50% to a concentration of 2.5 × 10^7^ – 3.0 × 10^7^ aggregates per millilitre and thereafter remained stable up until 24 h, suggesting that complete aggregation of amylin occurs within the first hour. Since it has been reported extensively that an incubation period of 20–24 h is sufficient for amylin to facilitate cytotoxicity,[12,13,15,18,19,70,73] it can be suggested that either the 100–150, 150–200 or 200–300 nm aggregates represents the toxic species of amylin.

Each of the 10 amylin derivatives was then individually co-incubated with disaggregated amylin and NTA was performed to evaluate their effect on amylin aggregation to the predominant sizes (Table 2). It appears that some of the non-methylated and methylated derivatives of amylin do exert a significant impact on the observed particle sizes at the 10 min and 24 h time intervals.

**Table 2.** Aggregate size distribution of amylin in the presence and absence of each of its derivatives.

	Concentration of aggregates (×10^7^ aggregates/mL)					
	100–150 nm		150–200 nm		200–300 nm	
Aggregates	10 min	24 h	10 min	24 h	10 min	24 h
A^#^	5.7	2.9	5.4	2.5	7.0	2.7
A + n1^#^	0.5*	0.9*	0.5*	1.1*	0.4*	0.8*
A + n2^#^	4.3	1.5*	2.5	1.5*	3.4	2.1*
A + n3^#^	2.0	3.4	1.2	2.1	1.4*	1.9*
A + n4^#^	3.8	1.7	2.3	1.8	5.1	1.7*
A + n5^#^	0.1*	2.4	0.1*	1.4*	0.5*	2.0*
A + m1^#^	4.0	2.2	2.2	2.8	2.4*	4.3
A + m2^#^	3.9	3.6**	2.4	7.9	2.6*	4.6
A + m3^#^	2.1	5.9	1.6	3.9	2.1*	4.7
A + m4^#^	2.7	4.1	1.5	2.8	1.6*	3.3
A + m5^#^	0.9*	1.6*	0.8*	1.3*	0.8*	1.4*

^#^A denotes amylin whilst A + n1, A + n2, A + n3, A + n4, A + n5, A + m1, A + m2, A + m3, A + m4 and A + m5 denote amylin plus each of its derivatives.

*Statistical analysis showed that this concentration of aggregates was significantly less (*p* < 0.05) than that of samples containing amylin only.

**Statistical analysis showed that this concentration of aggregates was significantly more (*p* < 0.05) than that of samples containing amylin only.

When disaggregated amylin was co-incubated with derivative n1, the concentration of aggregates in the three size ranges for the entire duration of the experiment was significantly less than that observed in samples containing disaggregated amylin only. It can thus be implied that this derivative could inhibit fibrillogenesis of amylin or conversely facilitate rapid aggregation of amylin into large aggregates that are out of the detection range of NTA since it has been reported that NTA can detect particles within a size range of 30–1000 nm.[63] It was previously shown that the 1–7 region of amylin is in a random coil conformation and could have a modulating effect on amylin aggregation, i.e. the amyloidogenic nature of amylin is increased if this region is excluded from the amylin sequence.[76] It thus appears that the derivative n1 which is an analogue of the 3–6 region of amylin could potentially delay fibril formation.

At the 10-min time interval, the concentrations of 100–150, 150–200 and 200–300 nm aggregates in the presence of derivatives n2 and n4 were not significantly different from that of samples containing disaggregated amylin only. However, after a 24-h period it was found that the concentrations of 200–300 nm aggregates were significantly less in samples containing disaggregated amylin and either derivative n2 or n4 than that of amylin only samples. It can therefore be construed that the derivatives n2 and n4 enhance amylin fibril formation to an aggregate size greater than 1000 nm. Analysis of samples containing disaggregated amylin and the derivative n3 illustrate that at the start of the experiment there is a small amount of aggregates of all size ranges which increases only marginally over 24 h. A similar trend was observed in samples containing disaggregated amylin and its derivative n5. Analysis of samples containing disaggregated amylin and either the derivative n3 or n5 suggest that these derivatives could also possibly increase or severely impede amylin aggregation, since at the start of the experiment there is a small amount of aggregates of all size ranges which increases only marginally over 24 h.

Shim et al. [20] proposed a model which demonstrates that residues 8, 13, 17, 25, 27 and 32 act as nucleation points for formation of β-sheets. The amylin derivatives n2, n3, n4 and n5 contain at least one of the proposed nucleation sites, thus implying that these derivatives have the inherent potential to facilitate formation of β-sheet structures thereby promoting the formation of fully aggregated amylin in a short space of time. In addition, these amylin derivatives also span regions of amylin that have previously been reported to form β-sheet structures.[13,17,19,22,65,76,89–92] Noteworthy and in keeping with this hypothesis, a previous report showed that fibril formation of full length amylin was enhanced in the presence of amylin derivatives n3 and n4 which are analogues of the 15–20 and 22–27 regions of amylin, respectively.[68,93] These previous findings would thus account for the potential of derivatives n2, n3, n4 and n5 to increase fibrillogenesis.

When compared to samples containing disaggregated amylin only, the derivatives m1, m2, m3 and m4 were found to have no significant effect on amylin aggregation, since after 24 h, the amount of 100–150, 150–200 and 200–300 nm aggregates formed in the presence of each of these derivatives were similar to or more than the number of aggregates present in samples containing disaggregated amylin only. This observation is supported by a previous observation that the introduction of *N*-methylated amino acids into peptides does not always result in inhibition of fibril formation.[94]

However, in the presence of derivative m5, there are significantly fewer amylin aggregates of all size ranges that form over the entire duration of the experiment when compared to samples containing amylin only, suggesting that this derivative either delays or increases amylin aggregation. Since the derivative m5 contains bulky methyl groups, it is most likely that these groups provide steric hindrance when the derivative binds to amylin, thereby destabilizing β-sheet structures. Thus, derivative m5 could possibly delay amylin aggregation.

A comparison of NTA-generated data to previously published reports implies that the amylin derivatives n1 and m5 inhibit fibrillogenesis whilst the derivatives n2, n3, n4 and n5 have the tendency to increase fibrillogenesis of amylin to an aggregate that is larger than 1000 nm. It is noteworthy that these 1000 nm aggregates are out of the detection range of NTA and could represent the fully aggregated form of amylin which has previously been reported to be non-toxic.[95–100] In the presence of amylin derivatives n1, n2, n3, n4, n5 and m5, the number of 200–300 nm amylin aggregates was significantly lower than in samples containing amylin only at 24 h. These derivatives were also previously reported to reduce amylin-mediated cytotoxicity by more than 40%.[73] Derivatives m1, m2, m3 and m4 were previously shown not to have an inhibitory effect on amylin-mediated cytotoxicity [73] and the concentration of 200–300 nm aggregates in samples containing disaggregated amylin and each of these derivatives were recorded to be similar to or more than the concentration of aggregates in samples containing disaggregated amylin only (≥2.7 × 10^7^ aggregates per millilitre). Based on these observations, it can thus be tentatively suggested that the 200–300 nm particle is the toxic species of amylin.

This study is the first to report on real-time monitoring of the aggregation of unconstrained amylin in solution. Furthermore, amylin was used in its unmodified form, thereby providing an insight into the behaviour of amylin under experimental conditions that are closely representative of those employed in mammalian cell line-based cytotoxicity studies. Data obtained also suggest that a critical concentration of 200–300 nm aggregates for amylin-mediated cytotoxicity is more than 2.1 × 10^7^ aggregates per millilitre. As mentioned previously, cell-based systems for screening potential inhibitors of amylin-mediated cytotoxicity can become cumbersome and time-consuming. Thus, after further optimization and verification studies, NTA could possibly be used as a quick screening technique for inhibitors of amylin-mediated cytotoxicity.

## Conclusions

This study demonstrated the appropriateness of SPR to be used in pursuit of the association and dissociation rates of amylin aggregation kinetics. SPR-derived data indicate that the association kinetics of amylin is similar to that of Aβ_1-40_ whilst it appears that amylin dissociates slower than Aβ_1–40_. In addition, NTA offers an attractive strategy as a rapid evaluative cell-free tool to assess inhibitors of amylin-mediated cytotoxicity. Moreover, NTA can also be used as a technique that caters for real-time monitoring of the amylin aggregation process whilst it continues unconstrained in solution. Importantly, the data from this study suggest that the size of toxic species of amylin is approximately 200–300 nm.
